# Cannabinoid Receptor *Type 2* Functional Variant Influences Liver Damage in Children with Non-Alcoholic Fatty Liver Disease

**DOI:** 10.1371/journal.pone.0042259

**Published:** 2012-08-23

**Authors:** Francesca Rossi, Giulia Bellini, Anna Alisi, Arianna Alterio, Sabatino Maione, Laura Perrone, Franco Locatelli, Emanuele Miraglia del Giudice, Valerio Nobili

**Affiliations:** 1 Department of Pediatrics, Second University of Naples, Naples, Italy; 2 Department of Experimental Medicine, Second University of Naples, Naples, Italy; 3 Department of Haematology-Oncology, Bambino Gesù Children's Hospital and Research Institute, Rome, Italy; 4 Liver Unit, Bambino Gesù Children's Hospital and Research Institute, Rome, Italy; 5 University of Pavia, Pavia, Italy; Sudbury Regional Hospital, Canada

## Abstract

Non-alcoholic fatty liver disease (NAFLD) comprises a spectrum of disease ranging from simple steatosis to inflammatory steatohepatitis (NASH) with different degrees of fibrosis that can ultimately progress to cirrhosis. Accumulating evidence suggests the involvement of the endocannabinoid-system in liver disease and related complications. In particular, hepatoprotective properties for Cannabinoid Receptor *type 2* (CB2) have been shown both through experimental murine models of liver injury and association study between a CB2 functional variant, Q63R, and liver enzymes in Italian obese children with steatosis.

Here, in order to clarify the role of CB2 in severity of childhood NAFLD, we have investigated the association of the CB2 Q63R variant, with histological parameters of liver disease severity in 118 Italian children with histologically-proven NAFLD.

CB2 Q63R genotype was assigned performing a TaqMan assay and a general linear model analysis was used to evaluate the association between the polymorphism and the histological parameters of liver damage.

We have found that whereas CB2 Q63R variant is not associated with steatosis or fibrosis, it is associated with the severity of the inflammation (*p* = 0.002) and the presence of NASH (*p* = 0.02).

Our findings suggest a critical role for CB2 Q63R variant in modulating hepatic inflammation state in obese children and in the consequent increased predisposition of these patients to liver damage.

## Introduction

Non-alcoholic fatty liver disease (NAFLD) comprises a spectrum of disease ranging from simple steatosis to inflammatory steatohepatitis (NASH) with different degrees of fibrosis that can ultimately progress to cirrhosis [1], [2]. Its prevalence is estimated to be between 20% and 30% in Western adults, rising to 90% in the morbidly obese. Of concern, NAFLD affects 2% to 10% of children and adolescents, this prevalence rising to 80% in obese children [3], [4].

Evidence that only a minority of patients with NAFLD progress to NASH suggests that disease progression is likely to depend on complex interplay between environmental factors and genetic predisposition [5]. Indeed, genetic variants in genes involved in energy balance, such as adiponutrin/patatin-like phospholipase domain-containing 3 (PNPLA3) and apolipoprotein C3 (APOC3), or in genes involved in inflammation, oxidative stress and fibrogenesis, such as SOD2, have been demonstrated to be associated with NAFLD and the severity of liver damage [6]–[8].

Cannabinoid receptors (CBs) are seven-transmembrane domain G protein-coupled receptors that bind phyto-derived, as well as endogenous cannabinoids, among which anandamide (AEA) and 2-arachidonoylglycerol (2-AG) are the best characterized. Two types of CBs are known to date, namely CB1 and CB2, respectively. CB1 are expressed at high levels in the central nervous system (CNS), whereas CB2 are found predominantly, but not exclusively, outside the CNS. CBs, together with the enzymes metabolizing AEA and 2-AG, form the endocannabinoid (EC) system [9], [10].

The EC system is involved in a wide range of regulatory functions and is now emerging as critical mediator of both acute and chronic liver injury [11]. This has been demonstrated by several preclinical models of nonalcoholic and alcoholic fatty liver, fibrosis, liver ischemia and cirrhosis showing that pharmacological modulation of EC system is effective in reducing liver injury [12]–[14].

Particularly, hepatoprotective and antifibrogenic properties have been recently suggested for CB2 receptors both through experimental murine models of liver injury and association study between a common functional variant of CB2, Q63R, with liver enzymes in a cohort of 438 Italian obese children with steatosis at ultrasound imaging [15]–[17].

In order to clarify the role of CB2 in severity of childhood NAFLD, in this study we have investigated the association of the Q63R CB2 variant with histological parameters of liver damage in a large series of Italian children with biopsy-proven NAFLD.

## Materials and Methods

### Patients

This study was carried on 118 consecutive untreated children and adolescents (41 males and 77 females) with biopsy-proven NAFLD who were referred to Bambino Gesù Children's Hospital between May 2006 and November 2009 [18], [19]. Secondary causes of steatosis such as alcohol abuse (more than 140 g/week), total parenteral nutrition, the use of drugs known to precipitate steatosis (e.g., valproate, amiodarone, and prednisone) and virus infections (Hepatitis A, B, C, D, cytomegalovirus, and Epstein-Barr) were excluded by appropriate tests. Autoimmune liver disease, metabolic liver disease, Wilson disease, and alpha-1-antitrypsin were ruled out with standard clinical and laboratory evaluations and/or through liver biopsy. All enrolled patients were Caucasians of Italian descent. The study was performed according to the recommendations of the ethics committee of Bambino Gesù Hospital and was conformed to the ethical guidelines of the 1975 Declaration of Helsinki. Informed consent from parents and assent from each patient were obtained.

Anthropometric and biochemical investigations, as well as evaluation of histology on liver biopsies were performed as previously described [18], [19]. Liver biopsy samples were at least 18 mm long and were read by a single liver pathologist who was unaware of the clinical and laboratory data of the patients. Biopsy samples were routinely processed (formalin- fixed and paraffin-embedded) and stained with hematoxylin and eosin and Van Gieson stains for the assessment of fibrosis and architectural changes.

The diagnosis of NASH was based on the pathologist's overall impression according to Kleiner et al. [20]. The main histological features commonly described for NAFLD, including steatosis, inflammation (both portal and lobular), hepatocyte ballooning, and fibrosis, were scored according to the scoring system for NAFLD as previously described [19]. Steatosis was graded on a four-point scale: 0) steatosis involving fewer than 5% of hepatocytes, 1) steatosis involving up to 33% of hepatocytes, 2) steatosis involving 33% to 66% of hepatocytes, and 3) steatosis involving more than 66% of hepatocytes. Lobular inflammation was also graded on a four point scale: 0) no foci, 1) fewer than two foci per field, 2) two to four foci per field, and 3) more than four foci per field. Hepatocyte ballooning was graded from 0 to 2: 0) no balloon cells, 1) few balloon cells, and 2) many/prominent balloon cells. The stage of fibrosis was quantified with a five-point scale: 0) no fibrosis, 1) perisinusoidal or periportal fibrosis [1a) mild, zone 3, perisinusoidal; 1b) moderate, zone 3, perisinusoidal; and 1c) portal/periportal], 2) perisinusoidal and portal/periportal fibrosis, 3) bridging fibrosis, and 4) cirrhosis.

Steatosis, inflammation, hepatocyte ballooning, and fibrosis were scored using the NAFLD Clinical Research Network (CRN) criteria [20]. Features of steatosis, lobular inflammation, and hepatocyte ballooning were combined to obtain the NAFLD activity score (NAS).

As recommended by a recent NASH CRN article [21], a microscopic diagnosis, based on overall injury pattern (i.e., steatosis, hepatocyte ballooning, and inflammation), as well as the presence of additional lesions (e.g., zonality of lesions, portal inflammation, and fibrosis), has been assigned to each case [22]. Accordingly, biopsies were subdivided into not steatohepatitis (not-SH) and definite steatohepatitis (definite-SH) subcategories [22].

### Genetic Analysis

After a written informed consent from parents and assent from children were obtained, genomic DNA was extracted from peripheral whole blood with a DNA extraction kit (Promega, Madison WI). Detection of the *CNR2* rs35761398 polymorphism (CAA/CGG), underlying the Q63R CB2 substitution, was performed by using a TaqMan Assay (Real Master Mix Probe, 5 PRIME, Germany).

Primers and probes were the following: Sense Primer 5′-GTGCTCTATCTGATCCTGTC-3′ and Anti-sense primer 5′-TAGTCACGCTGCCAATC-3′; AA-Probe 5′-CCCACCAACTCCGC-3′ and GG-Probe 5′-CCCACCGGCTCCG-3′ (PRIMM, Milan, Italy). Both polymerase chain reaction, performed according to the manufacturer's instruction, and post–polymerase chain reaction allelic discrimination through the measurement of allele-specific fluorescence were carried out on an ABIPRISM 9600 apparatus (Applied Biosystem, Foster City, CA). Random samples were confirmed by direct genotyping performing a PCR consisting in 94°C for 4 min followed by 31 cycles of 94°C for 30 s, 60°C for 30 s and 72°C for 30 s with Forward 5′-GAGTGGTCCCCAGAAGACAG-3′ and Reverse 5′-CACAGAGGCTGTGAAGGTCA-3′ primers. PCR products were analysed by using an ABI PRISM 3100 automated sequencer (Applied Biosystem, Foster City, CA). All primers were picked by using Primer3 software.

Patients were also screened, as previously described [23], for I148M PNPLA3 variant.

### Statistical Analysis

Results are expressed as means ± standard deviations. Mean values were compared by analysis of ANOVA, followed by Student–Newman–Keuls post hoc test. The differences in genotype and allelic frequencies and the association between the Q63R CB2 polymorphism with different clinical and liver histological parameters were evaluated by general linear model analysis including gender, age, waist circumference and HOMA-IR as covariates. Moreover we performed a multiple test including PNPLA3 genotype as covariate and then a multivariate analysis incorporating I148M PNPLA3 as well as Q63R CB2 variants for the whole cohort. Analyses were carried out by using Stat-Graph 3.0 software. In all cases *p*-values less than 0.05 were considered to be statistically significant.

## Results

To determine the influence of the Q63R variant on susceptibility to NASH, we examined whether this variant was associated with histological parameters of liver disease severity in a cohort of children with biopsy-proven NAFLD. Clinical and histological data of the children involved in the study are reported in Table 1.

**Table 1:** Clinical and Laboratory Characteristics of the 118 Italian Pediatric Patients with biopsy-proven NAFLD.

Feature	Mean ± SD or n (%)
**Female**	77 (65)
**Age (years)**	10.2±2.6
**BMI (centile)**	94.8±6.3
**Obesity**	107 (90.7)
**Waist Circumference (cm)**	84.7±10.3
**HOMA-IR index**	2.5±1.9
**ISI**	3.9±2.1
**Glucose (mg/dl)**	83.3±12.6
**Hypertension**	41 (35)
**IGT or diabetes**	59 (50)
**Total Cholesterol (mg/dl)**	162.3±34.8
**Triglycerides (mg/dl)**	110.8±65.6
**ALT (IU/ml)**	88.1±59.8
**AST (IU/ml)**	56.3±25.6
**Gamma-GT (IU/ml)**	32.4±19.9
**NASH (presence)**	53 (45)
**Inflammation stage (G0/G1/G2)**	3/92/23 (2.5/79/19.5)
**Steatosis stage (G0/G1/G2/G3)**	1/43/56/18 (1/36.5/47.5/15)
**Fibrosis stage (G0/G1/G2/G3)**	38/67/7/6 (32/57/6/5)
**CB2 Q63R variant (QQ/QR/RR)**	13/46/59 (11/39/50)
**PNPLA3 I148M variant (II/IM/MM)**	51-55-12 (43/47/10)
**CB2 Q63R/PNPLA3**	
**I148MQQ/II-IM-MM**	6-6-1
**QR/II-IM-MM**	20-23-3
**RR/II-IM-MM**	25-26-8

Values are expressed as means ± standard deviations. Abbreviations: BMI: Body Mass Index; HOMA-IR: homeostatic model of assessment of insulin resistance; ISI: insulin sensitivity index; IGT: Impaired Glucose Tolerance; ALT: alanine transaminase; AST: aspartate transaminase; Gamma-GT: Gamma-Glutamyl transferase; NASH: nonalcoholic steatohepatitis; G = grading.

As expected the great majority of the children was obese.

The frequency distribution of the Q63R CB2 SNP was in Hardy-Weinberg equilibrium. Heterozygosity for the at-risk allele encoding for R was observed in 39% of patients, and homozygosity was observed in 50%.

The Q63R genotype was not significantly associated with body mass index, lipid levels, insulin resistance, insulin sensitivity index, AST, ALT and gamma-GT levels (Table 2).

**Table 2:** Clinical and laboratory characteristics of the 118 Italian children with NAFLD stratified by the rs35761398 *CNR2* SNP encoding for the Q63R CB2 protein variant.

	*CNR2* rs35761398 variant			
	RR	QR	QQ	*p*
**N (%)**	59 (50%)	46 (39%)	13 (11%)	
**Age (years)**	10.2±2.7	10.2±2.6	9.9±2.5	0.92
**Female**	41 (69)	29(63)	7 (54)	0.52
**BMI (centile)**	95.0±6.3	95.5±4.9	91.7±9.4	0.15
**Waist Circumference (cm)**	85.1±10.9	84.7±9.8	82.6±9.5	0.73
**HOMA-IR index**	2.1±1.1	3.1±2.6	2.6±1.5	0.04
**ISI**	4.1±2.0	3.9±2.3	3.2±1.4	0.37
**Glucose (mg/dl)**	82.7±12.3	85.0±14.4	80.1±5.5	0.90
**Hypertension**	22 (37)	15 (32)	4 (31)	0.84
**IGT or diabetes**	29 (49)	22 (48)	8 (61)	0.67
**Total Cholesterol (mg/dl)**	164.6±35	157.1±33.3	170.4±39.5	0.37
**Triglycerides (mg/dl)**	113.9±71.9	98.7±56.1	139.2±60.2	0.12
**ALT (IU/ml)**	86.4±59.7	88.9±63.2	92.6±51.6	0.94
**AST (IU/ml)**	56.9±26.2	54.4±26.2	60.8±20.9	0.71
**Gamma-GT (IU/ml)**	33.9.±21.7	30.3±16.8	32.8±22.5	0.64

Values are expressed as means ± standard deviations or as numbers and percentages. Mean values are compared by analysis of ANOVA. GLM analysis including gender, age, waist circumference and HOMA-IR as covariates has been used to compare continuous variables. Abbreviations: BMI: Body Mass Index; HOMA-IR: homeostatic model of assessment of insulin resistance; ISI: insulin sensitivity index; IGT: Impaired Glucose Tolerance; ALT: alanine transaminase; AST: aspartate transaminase; Gamma-GT: Gamma-Glutamyl transferase.

A weak association was found with HOMA-IR index. Since the degree of insulin resistance is known to affect both severity of lobular inflammation and NASH, in the statistical analysis performed on liver histological data, beside age, sex and waist circumference, we added HOMA-IR index as covariate.

**Figure 1:**
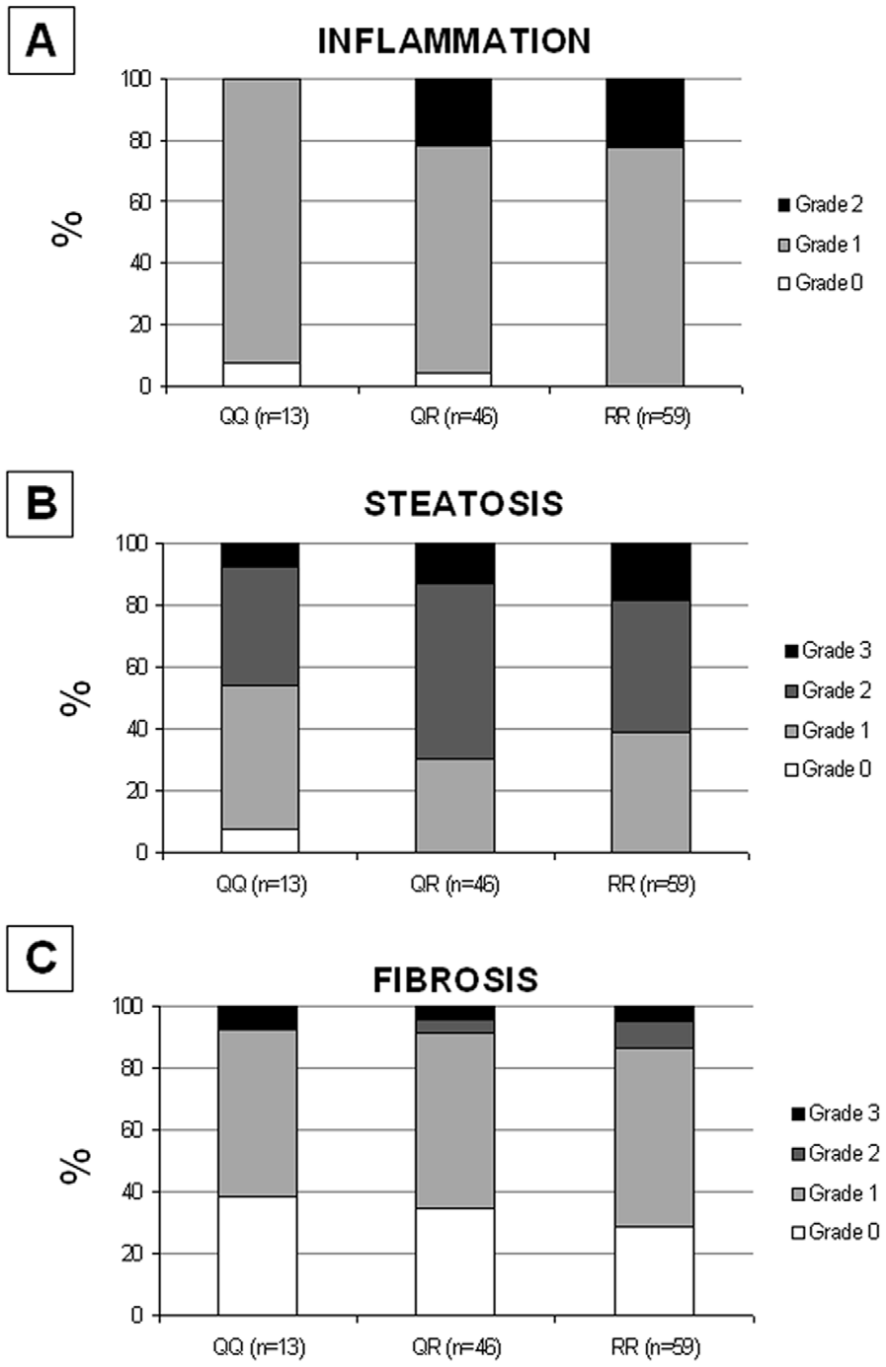
*CNR2* rs35761398 genotype and severity of liver lesions. Relationship between the *CNR2* rs35761398 genotype and the severity of A) inflammation (grades 0–2) (*p* = 0.0022); B) liver steatosis (grades 0–3) (*p* = 0.07); C) fibrosis (grades 0–3) (p = 0.24) in 118 children with NAFLD. P-values less than 0.05 have been considered statistically significant. Age, sex, waist circumference and HOMA-IR index have been used as covariates.

The Q63R genotype was strongly associated with the severity of inflammation. Grade 2 of inflammation was observed in 0 of 13 children with the QQ genotype, in 10 of 46 with the QR genotype (22%), and in 13 of 59 with the RR genotype (22%) (*p* = 0.0022; Fig. 1A).

The Q63R SNP was not associated with the severity of steatosis (grades 0–3) (*p* = 0.07; Fig. 1B), or of fibrosis (grades 0–3) (*p* = 0.24; Fig. 1C), neither with the ballooning (*p* = 0.93; not shown).

**Figure 2:**
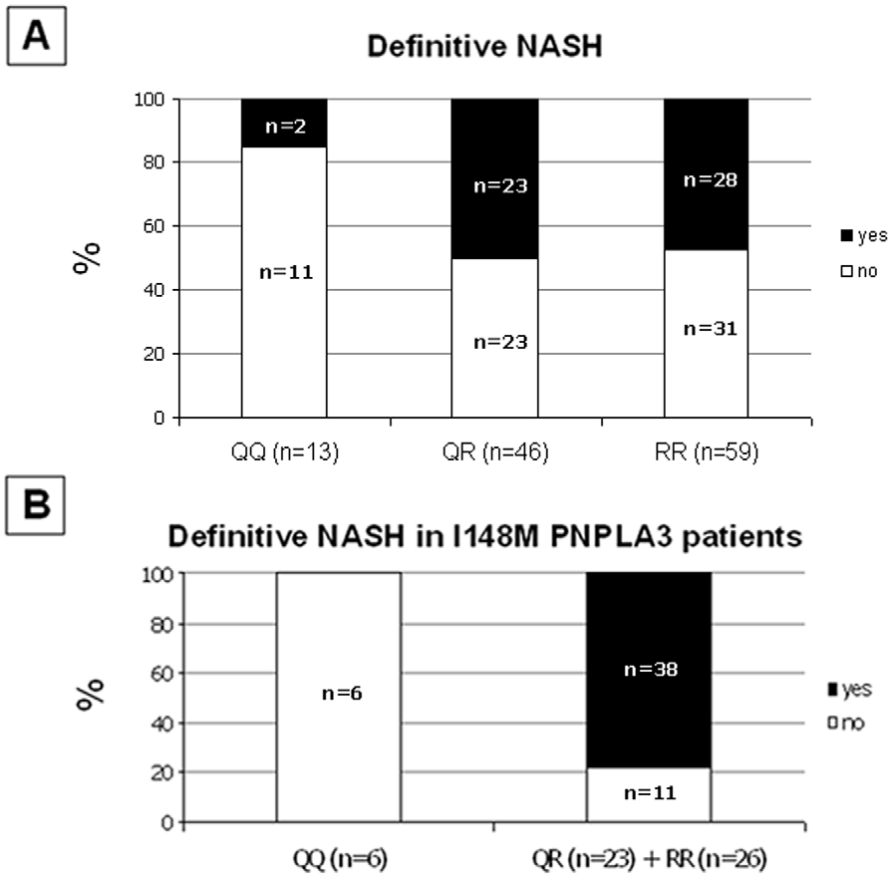
*CNR2* rs35761398 genotype and susceptibility to NASH. **A**) Relationship between the *CNR2* rs35761398 genotype and the presence of NASH in 118 children with NAFLD (*p* = 0.02). Fifty-three out of 118 patients show definitive NASH. Among these NASH subjects 2 were homozygous for the CB2 Q63 allele, 23 were CB2 Q63R heterozygous and 28 homozygous for the CB2 R63. **B**) Relationship between the *CNR2* rs35761398 genotype and the presence of NASH in 55 I148M PNPLA3 heterozygous children with NAFLD (*p* = 0.001). Among I148M PNPLA3 subjects 38 out of 55 show definitive NASH and were all QR or RR for the CB2 Q63R variant. Age, sex, waist circumference and HOMA-IR index have been used as covariates.

The prevalence of NASH according to the Q63R CB2 variant is shown in Figure 2A. Among children with NASH, 4% (2/53) had QQ genotype, 43% (23/53) showed QR genotype, whereas the remaining 53% (28/53) were RR homozygous (*p* = 0.02).

The relative odds ratios are shown in Table S1.

We also investigated the role of I148M PNPLA3 variant on NAFLD severity and, according to previous report [19], we found that subjects homozygous for I148 PNPLA3 variant were completely protected from NASH, whereas all M148 PNPLA3 homozygous patients showed definitive NASH. NASH was present in the 70% (38 out of 55) of I148M heterozygous subjects.

When the PNPLA3 genotype was included in the multivariate analysis the association between Q63R genotype and the severity of inflammation or the presence of NASH did not appear still significant (*p* = 0.069 and *p* = 0.12, respectively).

Performing a multivariate analysis incorporating both I148M PNPLA3 and Q63R CB2 variants, as well as age, sex, waist circumference and HOMA-IR, the association with inflammation and steatosis, driven by the PNPLA3 genotype, was significant (*p*<10^−4^), whereas the association with fibrosis was not (*p* = 0.08) (Table S2).

When we stratified the sub-group of 55 children I148M PNPLA3 heterozygous for the Q63R CB2 variant, we found that the presence of NASH among these patients was significantly associated to the presence of the R63 CB2 allele (*p* = 0.001; Fig. 2B) and that the odds ratio-associated *p* value was 0.0001.

## Discussion

Considering that only a portion of patients with NAFLD will progress from simple fatty liver (steatosis) to inflammation (steatohepatitis), fibrosis and ultimately cirrhosis, it appears as an important goal to understand the mechanisms leading to this progression [24]. Data from literature evidence that this progression is triggered by a number of factors, such as oxidative stress, endoplasmic reticulum stress, fatty acid lipotoxicity, immune system activation, inflammatory cytokine production, possibly able to determine the different spectrum of disease [25].

It has been shown in rodents that the setting of simple hepatic steatosis is associated with a chronic hepatic inflammatory state, due to increased activation of the nuclear factor κB (NF-κB) transcription factor which, in turn, functioning as a pro-inflammatory switch, induces the transcription of inflammatory cytokines, including TNFα and IL-6, in the hepatocytes and in the Kupffer cells [26].

The production of these inflammatory cytokines seems to play a major role in the further step from steatosis to steatohepatitis progression.

The well-know effects of CB2 receptor on both peripheral and central inflammatory response and mainly its ability to modulate both activation of NF-κB and the production of TNFα and IL-6 appear of particular interest in the context of the study we have performed. Experimental studies have, in fact, demonstrated that CB2 stimulation is able to inhibit NF-κB translocation into the nucleus, interfering with the phosphorylation of its activator, and to decrease both TNFα and IL-6 production, enhancing the expression of their synthesis inhibitor, the anti-inflammatory cytokine IL-10 [27]–[29].

In 118 Italian children with biopsy-proven NAFLD, the CB2 variant carrying an arginine at codon 63 was not associated with steatosis, but was associated with the severity of the NAFLD inflammatory component, this increasing the risk for NASH appearence. In a previous study [16] we have shown, in a group of obese children with liver steatosis at ultrasound imaging, the presence of an association between the Q63R CB2 variant and liver enzymes. In the present work we did not replicated this result. The reason for this apparent discrepancy resides in the criteria used, in this study, to enrol the patients. They are patients that underwent liver biopsy and they show particularly high levels of liver enzymes. This homogeneous increased level of ALT and AST has not allowed to highlight the role of CB2 polymorphism in modulating liver enzyme levels.

It has been recently demonstrated that the CB2 exhibiting an arginine at codon 63 (R63) shows a reduced function when activated by an endogenous cannabinoid ligand, compared to the CB2 variant with the glutamine at the same codon (Q63) [30], [31]. Therefore, the possibility exists that the protective modulating effect exerted by a completely active CB2 system on liver inflammation may be, at least partially, lost in the individual carrying the allele encoding for CB2 R63.

Nevertheless the lack of association of CB2 polymorphism with fibrosis may simply reflect the young age of the cohort (10.2±2.6) as they have no sufficient time for the disease to progress.

Recently, the functional variant I148M of PNPLA3, a gene likely involved in hepatic triglyceride hydrolisis, has been associated with steatosis severity and with the presence of NASH in the same cohort here studied [19]. In fact, whereas I148 PNPLA3 homozygous patients showed very mild and often uncomplicated steatosis, heterozygous and homozygous M148 patients were strongly at risk of severe steatosis. Attempting to delineate the role of genetic predisposition in NAFLD severity, we suggest that PNPLA3 and CB2 may act in two different, consecutive hits producing liver damage. PNPLA3 polymorphism primitively predisposes to liver steatosis which in turn activates the cascade of events leading to liver inflammation. CB2 polymorphism may work only later, when steatosis is already present and NF-κB is up-regulated, modulating the transcription of several inflammatory cytokines.

Accordingly, stratifying CB2 polymorphism on I148M PNPLA3 variant, we have noticed that the effect of R63 CB2 variant on NASH appearance was emphasized in the sub-group of patients carrying the M148 PNPLA3, which is associated to severe steatosis.

The data reported in this work, therefore, may be explained by the existence of different pathways contributing to liver damage progression in NAFLD, suggesting an important role of the inflammatory component in its pathogenesis and in its progression towards more severe liver disease.

Considering that NAFLD is the most common cause of chronic liver disease in children and given the highly heritable nature of this disease [32], our results contributing to a better understanding of the genetic factors involved in NAFLD severity may enable improved identification of NAFLD children at risk for disease progression and may help in the identification of new molecular targets for pharmacological treatments.

## Supporting Information

Table S1 Multivariate analysis incorporating both I148M PNPLA3 and Q63R CB2 variants, as well as age, sex, waist circumference and HOMA-IR.
(DOC)

Table S2 Odds ratio for liver disease features with respect the Q63R CB2 variant. According to the data from the multivariate analysis, odds ratio associated *p*-values were significant only for the presence of NASH. It worth of notice that the odds ratio for inflammation risk with respect the CB2 variant is 0 for the QQ allele, since none of QQ subjects show a grade 2 of inflammation.
(DOC)
