# Supplementary material for: Cannabinoid Receptor Type 2 Functional Variant Influences Liver Damage in Children with Non-Alcoholic Fatty Liver Disease
Source: PLoS One. 2012 Aug 23;7(8):e42259. doi: 10.1371/journal.pone.0042259 (PMC3426511; doi:10.1371/journal.pone.0042259)
Supplement: Table S1 — Multivariate analysis incorporating both I148M PNPLA3 and Q63R CB2 variants, as well as age, sex, waist circumference and HOMA-IR. (DOC) [file pone.0042259.s001.doc]

#### Table S1

**Odds ratio for liver disease features with respect the Q63R CB2 variant**

| **Features presence** | **Odds Ratios** | *Odds Ratio* | *CI 95%* | ***p*** |
| --- | --- | --- | --- | --- |
| FIBROSIS G0 *vs* G1+G2+G3 | QQ vs (QR+RR) | 0.73 | 0.22-2.41 | 0.61 |
| **STEATOSIS** |  | 0.46 | 0.14-1.49 | 0.19 |
| **BALLONING** |  | 1.14 | 0.35-3.63 | 0.82 |
| **INFLAMMATION**  mild *vs* severe (grade 0+1 *vs* grade 2 |  | 0* | - | - |
| NASH |  | 0.19 | 0.04-0.91 | **0.02** |

* none of QQ subjects shows a grade 2 of inflammation
