# Supplementary material for: Cannabinoid Receptor Type 2 Functional Variant Influences Liver Damage in Children with Non-Alcoholic Fatty Liver Disease
Source: PLoS One. 2012 Aug 23;7(8):e42259. doi: 10.1371/journal.pone.0042259 (PMC3426511; doi:10.1371/journal.pone.0042259)
Supplement: Table S2 — Odds ratio for liver disease features with respect the Q63R CB2 variant. According to the data from the multivariate analysis, odds ratio associated p-values were significant only for the presence of NASH. It worth of notice that the odds ratio for inflammation risk with respect the CB2 variant is 0 for the QQ allele, since none of QQ subjects show a grade 2 of inflammation. (DOC) [file pone.0042259.s002.doc]

# Supplementary Data, S2

# General Linear Models

Number of dependent variables: 1

Number of categorical factors: 2

### Q63R CB2 and I148M PNPLA3

Number of quantitative factors: 4

### Sex; Age; Waist Circumference; HOMA-IR

## Analysis of Variance for STEATOSIS

| *Source* | *Sum of Squares* | *Df* | *Mean Square* | *F-Ratio* | *P-Value* |
| --- | --- | --- | --- | --- | --- |
| Model | 34,9005 | 8 | 4,36256 | 20,89 | **0,0000** |
| Residual | 22,3409 | 107 | 0,208793 |  |  |
| Total (Corr.) | 57,2414 | 115 |  |  |  |

### Analysis of Variance for INFLAMMATION

| *Source* | *Sum of Squares* | *Df* | *Mean Square* | *F-Ratio* | *P-Value* |
| --- | --- | --- | --- | --- | --- |
| Model | 8,18133 | 8 | 1,02267 | 7,61 | **0,0000** |
| Residual | 14,3704 | 107 | 0,134303 |  |  |
| Total (Corr.) | 22,5517 | 115 |  |  |  |

# Analysis of Variance for FIBROSIS

| *Source* | *Sum of Squares* | *Df* | *Mean Square* | *F-Ratio* | *P-Value* |
| --- | --- | --- | --- | --- | --- |
| Model | 7,73033 | 8 | 0,966292 | 1,82 | 0,0809 |
| Residual | 56,7783 | 107 | 0,530638 |  |  |
| Total (Corr.) | 64,5086 | 115 |  |  |  |
